# Chronic active Epstein-Barr virus infection of T-cell type, systemic form in an African migrant: case report and review of the literature on diagnostics standards and therapeutic options

**DOI:** 10.1186/s12885-018-4861-0

**Published:** 2018-10-03

**Authors:** Maxi Wass, Marcus Bauer, Roald Pfannes, Kerstin Lorenz, Andreas Odparlik, Lutz P Müller, Claudia Wickenhauser

**Affiliations:** 1 Department of Hematology and Oncology, University Hospital Halle, Germany, Ernst-Grube-Str. 40, Halle, 06120 Germany; 2 Department of Pathology, University Hospital Halle, Germany, Magdeburger Str. 2, Halle, 06112 Germany; 3 Department of General Visceral, Vascular and Endocrine Surgery, University Hospital Halle, Germany, Ernst-Grube-Str. 40, Halle, 06120 Germany; 4 Department of Radiation Therapy and Nuclear Medicine, University Hospital Halle, Germany, Ernst-Grube-Str. 40, Halle, 06120 Germany

**Keywords:** Epstein-Barr virus, Chronic active Epstein-Barr virus infection, T/NK-cell lymphoproliferative disease, Adults, Western countries, Diagnostic standards, Treatment

## Abstract

**Background:**

Chronic active Epstein-Barr virus (EBV) infection (CAEBV) of the T-/NK-cell type, systemic form is a rare and potentially life-threatening illness caused by persistent EBV infection. The highest incidence is found in children and adolescents with increased frequency among Asians and Native Americans, while the disease is uncommon in Western countries. Typically patients present with unspecific symptoms, like fever, lymphadenopathy, hepatosplenomegaly and liver dysfunction. Due to fatal complications including hemophagocytic syndrome, coagulopathy, multiple organ failure and development of EBV-positive lymphoproliferative disease (LPD) or lymphoma early diagnosis is critical for successful treatment. However, in consequence of the lack of experience due to the low incidence in Europe, a broad spectrum of clinical manifestations and a particularly unexpected group of patients, diagnosis can be challenging. Inhere we describe the clinicopathological findings of an African adult with CAEBV associated LPD with a brief review of the literature.

**Case presentation:**

A 42-year-old African man with fever, enlargement of the spleen and a suspected epileptic seizure was referred to our hospital. Diagnostic testing repeatedly revealed a massive EBV-DNA load in peripheral blood. Whole-body PET-CT-scan presented a strong uptake at multiple bone marrow sites, the thyroid and the adrenal glands. Histopathological analysis of bone marrow and thyroid gland revealed a highly proliferating, atypical and predominantly intravascular cytotoxic T-cell population with intracellular EBV-encoded RNA. Clonality analysis revealed the presence of polyclonal T-cell-receptor. Based on these findings a CAEBV of the T-/NK-cell type, systemic form was diagnosed. Subsequent therapy including three cycles of chemotherapy with cyclophosphamide, doxorubicin, vincristine and prednisolone resulted in decreased EBV load, clinical improvement and ongoing complete remission.

**Conclusion:**

Adult-onset CAEBV of T/NK-cell type usually comprises a poor prognosis and is extremely rare in Western countries. Therefore, our case highlights the need for a clinical awareness of this disease in patients with systemic illness and for a comprehensive multidisciplinary diagnostic approach to facilitate diagnosis. Treatment options include antiviral drugs, immunosuppressive agents and systemic chemotherapy with or without allogeneic stem cell transplantation. Given the limited data these options need to be decided upon in each patient individually considering severity of the disease, comorbidities and response.

## Background

Chronic active Epstein-Barr virus (EBV) infection (CAEBV) of T/NK-cell type, systemic form, which belongs to the broad spectrum of EBV-positive lymphoproliferative disorders (LPD) [1], is a rare and life-threatening illness with highest incidence among children and adolescents from Asia and South America [2, 3]. The symptoms are often unspecific and include unexplained fever, lymphadenopathy and/or hepatosplenomegaly [3, 4]. Potential complications include hemophagocytic syndrome, hepatic failure, coagulopathy, sepsis and multiple organ failure [3–5]. A standard treatment has not been yet established. Antiviral therapy is generally ineffective and although chemotherapy is an important treatment, allogeneic stem cell transplantation (SCT) usually remains the only curative option [6, 7]. Thus, early diagnosis is critical for a successful treatment. However, given the variable clinical presentation and histopathological features diagnosis can be challenging.

## Case presentation

Here we present the case of a 42-year-old man from Western Africa who had been immigrating to Germany recently. Initial admission was due to a seizure and a history of wasting and worsening health condition for six months. Upon admission he presented with recurrent fever, hepatosplenomegaly and acute kidney failure. Laboratory test results revealed a mild pancytopenia (leukocytes 2.28 × 10^9^/L, hemoglobin 6.2 g/dL, platelets 113 × 10^9^/L), increased LDH (702 IU/L) and nephrotic syndrome (proteinuria 12 g/d). Cranial magnet resonance imaging (MRI) presented no pathological findings. Bone marrow histology identified single atypical cells in intravascular position. Due to the rarity of these atypical cells further specification of their nature including immunohistochemistry and molecular techniques was not possible. Cytogenetic testing of the bone marrow aspirate but not the peripheral blood revealed a complex karyotype in single metaphases.

Ultrasound of the abdomen showed hepatosplenomegaly but no lymph node swelling. Further diagnostic approaches included computed tomography (CT) scan of the chest which revealed atypical pulmonary infiltrates and bilateral hilar lymphadenopathy. Transbronchial biopsy was negative for infectious agents, sarcoidosis or malignancy. As bronchial lavage was positive for Aspergillus antigen, the patient was treated with Voriconazole. However, the assumption of pulmonary aspergillosis did not explain all of the patient’s symptoms. Further diagnostic approaches included high positive proteinase 3 titer (1:135) and therefore granulomatosis with polyangiitis was suspected. However, given low platelet counts biopsy of the kidney was not performed.

**Fig. 1:**
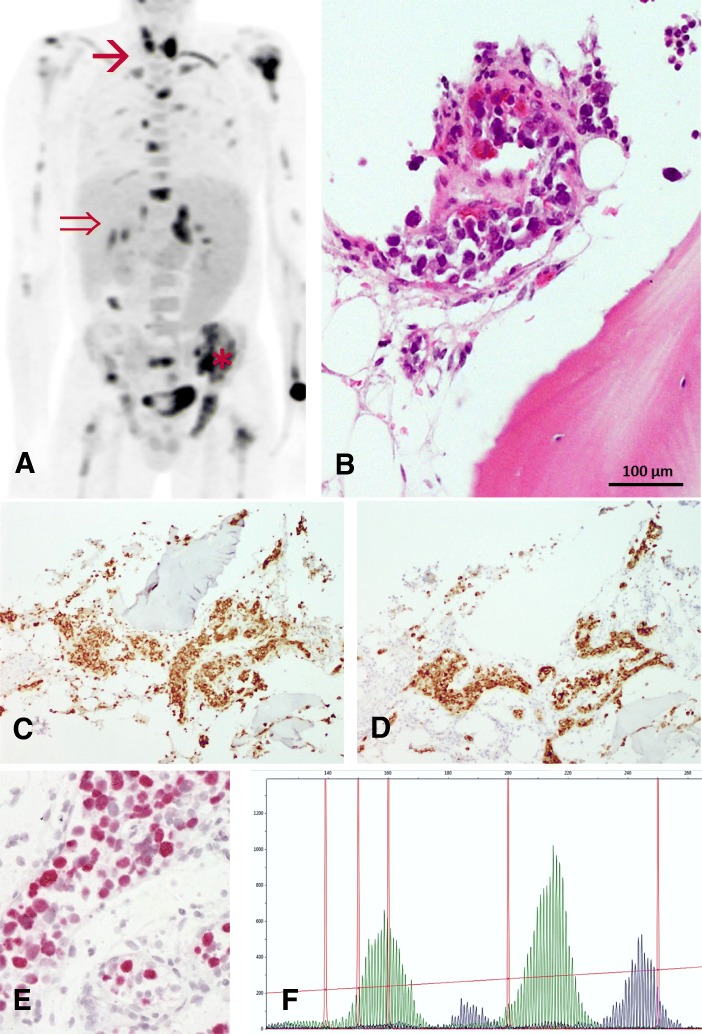
Detection of Chronic Active Epstein-Barr-Virus infection associated T-cell-lymphoproliferative disease. **a** PET-CT showed suspect glucose uptake in the thyroid (➔), liver (⇒), as well as in the iliac crest (_*_). **b** Targeted bone marrow biopsy revealed aggregates of intravascular pleomorphic cells (HE, × 200). Immunohistochemistry confirmed positivity for CD3 (**c**) and Perforin (**d**). Furthermore EBER-CISH demonstrated active EBV infection within these T-cells (**e**). **f** Subsequently T-cell receptor clonality analysis resulted a polyclonal T-cell population (vγ1–8, vγ10)

Considering the clinical symptoms, the patient was treated with high-dose steroid burst. However, upon that the patient’s condition worsened rapidly with further loss of weight and intermittent high fever, despite antibiotic and antifungal treatment. Thus, steroids were withdrawn. Infectious disease testing, including human immunodeficiency virus, Tuberculosis, Schistosomiasis, Malaria, and Leishmaniosis were negative, expect of previous Hepatitis B and EBV infection (anti EBV VCA IgM-ELISA negative, anti EBV VCA-ELISA IgG 11 E/ml; anti EBNA IgG-ELISA 14 E/ml). While no signs of chronic HBV infection were seen, EBV DNA in the peripheral blood reached high levels (2.8 × 10^6^ copies/mL). Along with the clinical symptoms CAEBV was therefore assumed, without evidence of previous immune deficiency. Positron emission tomography (PET)-CT revealed a high glucose uptake in the thyroid, liver, adrenal gland and in the examined bone areas (Fig. 1a).

**Fig. 2:**
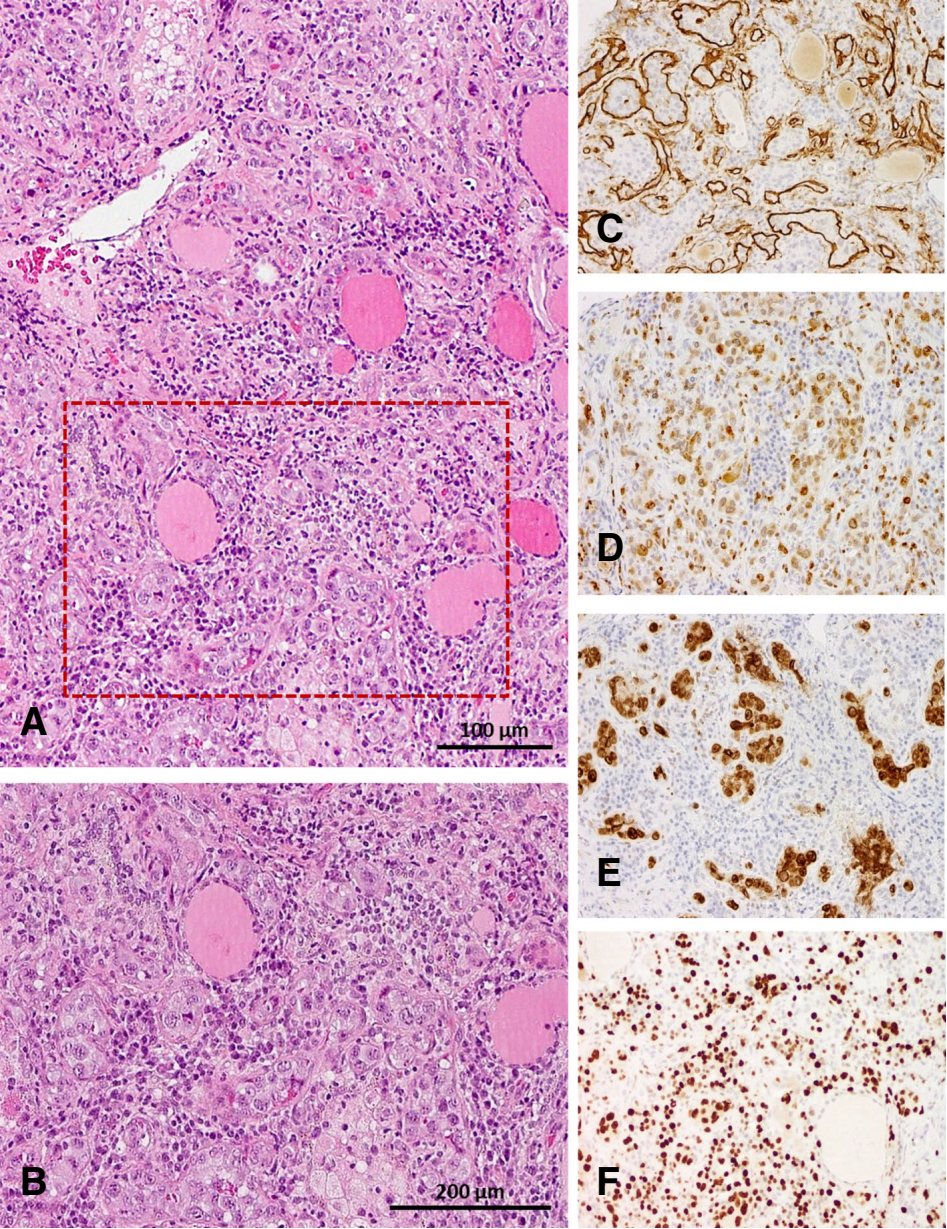
Thyroid gland involvement. **a** and **b** Biopsy of thyroid gland revealed large aggregates of intravascular located middle-sized highly pleomorphic cells on HE stained slides (× 100 and × 200). The implementation of CD34 immunohistochemistry (**c**) ensured intravascular localization of these cells, characterized by positivity for CD3 (**d**) and Perforin (**e**). Further Ki67 staining showed high proliferative activity (**f**)

Based on PET-CT findings a targeted biopsy of the bone marrow and the thyroid gland were performed to facilitate a definite diagnosis. Histological evaluation of the bone marrow biopsy now revealed a hyperplastic hematopoiesis with prominent erythropoiesis and left shifted myelopoiesis. In addition, in this biopsy conventional histomorphology (Fig. 1b), underlined by immunohistochemical findings revealed small aggregates of partly intravascular localized, highly proliferated (Ki67 90%) middle–sized lymphoid cells with coexpression of CD3 (Fig. 1c) and perforin (Fig. 1d) but missing expression of CD4 and CD8. Additional findings included negativity of the atypical T-cells for CD34, TdT, CD20 and CD56. Performing EBER-in-situ-hybridization active EBV infection within these T-cells was detected (Fig. 1e). Parallel examination of thyroid gland tissue provided large aggregates of the identical middle-sized highly pleomorphic and proliferated T-cell population with consecutive displacement of thyroid follicles (Fig. 2a-f). T-cell receptor clonality analysis by amplification of Vɣ1–11-region und multiple Jɣ-regions of T-cell receptor-gamma-gene provided a polyclonal pattern (Fig. 1f). Altogether, histological findings along with flow cytometry analysis and molecular data excluded diagnosis of T-cell lymphoma or lymphoblastic leukemia. As hemophagocytic lymphohistiocytosis (HLH) is often associated with CAEBV the clinical criteria for HLH were evaluated but were not fulfilled. Moreover, no typical findings of HLH were seen in the bone marrow biopsy. According to the 2017 WHO classification diagnosis of CAEBV of T/NK-cell type, systemic form was finally made [8].

**Fig. 3:**
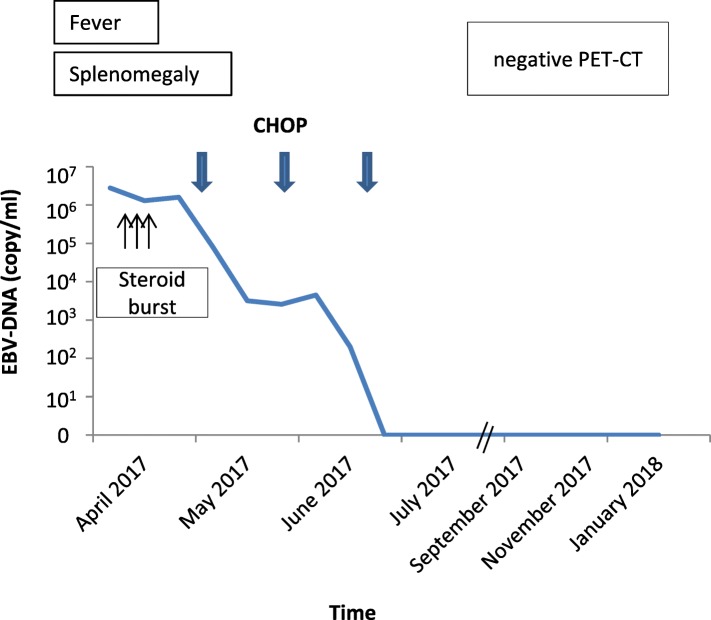
Clinical course. The graph shows the significant drop of Epstein-Barr viral load in the peripheral blood after initiation of chemotherapy. After 3 cycles of CHOP chemotherapy PCR for EBV-DNA was negative. In addition, PET-CT scan revealed no glucose-uptake of initial positive lesions. CHOP: cyclophosphamide, doxorubicin, vincristin and prednisolon

Based on the data by Sawada et al. [9], the patient was then treated with methylprednisolone and cyclophosphamide as cooling down step to reduce the high viral load. Given the persisting renal failure we refrained from immunosuppression with cyclosporine A. In the further course the patient developed an epileptic seizure and clinical symptoms of meningitis. To clarify whether these symptoms were related to CAEBV or EBV-associated T-cell LPD a lumbar puncture was performed and cranial MRI was repeated. Indeed, cerebrospinal fluid was positive for EBV DNA but did not include atypical T-cells. Cranial MRI now showed typical radiological signs for viral meningitis. We regarded this as a clinical progression and initiated chemotherapy with cyclophosphamide, doxorubicin, vincristine and prednisolone (CHOP). Despite its limited activity for EBV-infection we also started treatment with foscavir. Upon this and 2 cycles of CHOP the EBV load decreased remarkably from 2.8 × 10^6^ copies/mL to < 200 copies/mL. An additional cycle of CHOP was administered and the patient remained in complete remission so far (Fig. 3).

## Discussion and conclusion

EBV infection usually causes polyclonal or monoclonal proliferations of B-cells, typically in immunocompromised hosts. In very rare cases, mostly in Asia or South America, EBV-associated LPD develop, that include polyclonal, oligoclonal and monoclonal proliferations of T-cells and/or NK-cells [3–5]. While EBV-associated LPD of B-cells mainly occurs in older patients, EBV-associated LPD of T/NK-cells affects predominantly younger individuals [4, 10, 11]. LPD of T/NK-cells encompasses different clinical entities such as CAEBV, EBV-associated HLH, hypersensitivity to mosquito bites and severe-type hydroa vacciniforme [2, 3, 12]. Revised diagnostic criteria for CAEBV encompass EBV-related illness of greater than three months duration, increased EBV DNA (≥ 10^2,5^ copies/μg) in peripheral blood or RNA in affected tissue as well as abnormally high levels of EBV antibodies [3]. A proposed clinicopathologic classification of CAEBV associated LPD of T/NK-cells based on pathologic and molecular data differentiates three categories: polymorphic LPD without clonal proliferation (A1), polymorphic LPD with clonality (A2) and monomorphic LPD with clonality [1, 5]. Based on this categorization the here presented case has to be classified as polymorphic LPD without clonal proliferation.

Due to its rarity in Western Europe and its frequently unspecific morphological signs as well as the broad spectrum of clinical manifestations CAEBV diagnosis can be challenging. The here presented patient presented with persistent high-grade fever, pancytopenia and splenomegaly. There was no lymph node swelling and transaminases were in the normal range. The determinant findings in our case were the high viral EBV load in the peripheral blood and the PET-CT scan facilitating targeted biopsy with conclusive histopathological analysis. The biopsies revealed the atypical T-lymphocyte population in predominantly intra- and paravascular position and positive EBER-in-situ-hybridization. The intravascular localization and the high glucose uptake in PET-CT is unusual [13], however immunohistochemistry staining and T-cell receptor clonality analysis providing a polyclonal population clearly ruled out aggressive lymphomas, including extranodal NK/T-cell lymphoma/leukemia, EBV-positive peripheral T-cell lymphoma or intravascular large B-cell lymphoma. The detected complex karyotype is rare, but has previously described in CAEBV related T-cell LPD [10] and might be a result of subclonal expansion of EBV-infected T-cells rather than infectious.

Treatment options of CAEBV-related T-cell LPD include antiviral drugs, immunosuppressive agents and systemic chemotherapy with or without allogeneic SCT. It is important to mention that antiviral therapy alone generally is ineffective and immunosuppressive agents like corticosteroids and cyclosporine often only temporarily reduce symptoms [6]. Chemotherapeutic drugs may be useful but include a high risk of disease progression and relapse [7, 9]. Especially patients with fulminant clinical course may not benefit from chemotherapy alone. Allogeneic SCT has been reported to be a successful option in some cases but depends on donor eligibility and is associated with high treatment related mortality [6, 7, 9]. Since there are no evaluated prognostic risk factors for CAEBV treatment remains individual. The here presented case suggests that patients with low antibody-titer, polymorphic and polyclonal LPD (A2) may be sufficiently treated with CHOP like regimens.

In conclusion, adult-onset CAEBV associated T-cell LPD usually shows a poor prognosis and is extremely rare in Western countries. Early diagnosis is critical for a successful treatment. However, in consequence of the lack of experience due to the low incidence in Europe, a broad spectrum of clinical manifestations and a particularly unexpected patient group diagnosis can be challenging. Therefore, our case highlights the need for a clinical awareness of this disease in patients with systemic illness and unclear symptoms as well as for a comprehensive multidisciplinary diagnostic approach to facilitate diagnosis. Given the limited data, treatment options like systemic chemotherapy and allogeneic SCT need to be carefully evaluated and decided upon in each patient individually considering severity of the disease, comorbidities and response.

## Abbreviations

CAEBV: Chronic active EBV infection
CHOP: Cyclophosphamide, Doxorubicin, Vincristine, Prednisolone
CT: Computed tomography
EBV: Epstein-Barr virus
HLH: Hemophagocytic lymphohistiocytosis
LPD: Lymphoproliferative disorders
MRI: Magnet resonance imaging
PET: Positron emission tomography
SCT: Stem cell transplantation
